# The Chinaman vs. the Sanitarian

**Published:** 1886-08

**Authors:** 


### *THE CHINAMAN VS. THE SANITARIAN.*

The BUFFALO MEDICAL AND SURGICAL JOURNAL says: "We welcome the *Southern California Practitioner* in our list of exchanges. This is a new journal, published in Los Angeles, and devoted to general medicine, special attention being paid to the climatology of Southern California. The journal is neat in appearance, and ably edited. We have but one criticism to offer: the editors seem to have been imbued with an excessive amount of the anti-Chinese spirit."

The New York *Medical Record*, in the course of an editorial on "The Chinaman from a Medical Point of View," in which it quotes from the *Southern California Practitioner*, says: "About the same thing can be said of the lower class of Italians, and of Russian and Polish Jews, which are now being poured upon the Eastern shore." We believe that the editors of the BUFFALO JOURNAL and of the *Medical Record* having doubtless spent their lives on the Atlantic side of the continent, are perfectly capable of judging the immigrants who flock to their great cities, and we ask that we, who have spent our professional lives on the Pacific coast, be adjudged capable of giving an unbiased opinion of the Jew, Gentile and Heathen who locate among us. Still, fearing that our editorial brethren may yet consider us prejudiced, we refer them to the following statement from that very conservative Congressman, Mr. Milliken, of Maine:

"Chinatown," he says, "occupies four blocks in the heart of the city. You break off abruptly from the rest of the city when you go into this quarter. You see none but Chinese. They are as utterly separated from the whites, except for the police and occasional visitors like ourselves, as though they were in the heart of the Celestial empire. The Chinese are inveterate gamblers, and their gaming shops are thick along the streets. You see crowds of men, a very few women, and no children. I don't think I ever saw a dozen of children in Chinatown. There are no family organizations among these people, and the Chinese women, who confine their relations to men of their own race, are not looked upon as prostitutes by the Chinese.

"We went down into the Chinese dens, three stories under the ground. In these dives, which are reached through narrow, dark, filthy passages, we found crowds of Chinamen smoking opium. The stench was almost unbearable to American nostrils. I believe

Chinatown could cause a pestilence in almost any American city except San Francisco, where the winds from the ocean sweep across the city at certain hours, and clear out the poisonous atmosphere. * * * Their filthy habits and their grossly immoral sexual relations make them a blot and scourge to the city of San Francisco. They have no families or homes like other people." * * *

Mr. Milliken then proceeds to advocate the total prohibition ot Chinese immigration.

What he says of Chinatown in San Francisco, is true of Los Angeles, and every other Pacific coast city, in proportion to population. While we wish the editors of the BUFFALO JOURNAL a far better fortune, yet it might be a good thing if a Chinatown, with its opium joints, its prostitutes, its gambling dens, its joss-houses and its lodging-houses, was located for one year in the heart of the prosperous lakeside metropolis. We trust, then, that the "anti-Chinese spirit" of the Buffalo editor would not be "excessive."—*Southern California Practitioner.*

